# Wideband Substrate Integrated Waveguide Chip Filter Using Spoof Surface Plasmon Polariton

**DOI:** 10.3390/mi13081195

**Published:** 2022-07-28

**Authors:** Dongzhe Pan, Bin You, Xuan Wen, Xungen Li

**Affiliations:** 1 The Key Laboratory of RF Circuits and Systems of Ministry of Education, Microelectronics CAD Center, School of Electronics and Information, Hangzhou Dianzi University, Hangzhou 310018, China; pdz@hdu.edu.cn (D.P.); lixg@hdu.edu.cn (X.L.); 2 The School of Information Engineering, Hangzhou Dianzi University, Hangzhou 310018, China; fyrrrr@163.com

**Keywords:** substrate integrated waveguide (SIW), spoof surface plasmon polaritons (SSPPs), integrated passive device, through-dielectric capacitor (TDC)

## Abstract

This article presents a novel wideband bandpass filter based on the integration of a substrate integrated waveguide (SIW) and a spoof surface plasmon polariton (SSPP). An SIW cavity with periodic arrays of meander-slot units is etched on the top metallic layer to achieve the characteristics of a multi-order filter with good performance. The passbands can be flexibly selected by varying the geometric parameters of the SIW and SSPP to adjust the lower and upper sidebands independently. Using a redistribution layer (RDL) process, a novel 3D capacitive interconnection called a through-dielectric capacitor (TDC) is proposed and collaboratively designed with an interdigital capacitor to achieve capacitive source-load cross-coupling. The proposed filter has a center frequency of 60 GHz, with a wide 3-dB fractional bandwidth of about 45.8%. The improved simulated sideband suppression has a 30 dB rejection at 40 GHz and 75.4 GHz, corresponding to a 30-dB rectangular coefficient of 1.28.

## 1. Introduction

With the availability of 60 GHz unlicensed frequency bands and millimeter-wave spectrum for fast data transmission, wireless systems are increasingly operating at higher frequencies. Meanwhile, filters are key front-end components in communication systems, and higher transmission rates must be supported by wider bandwidths and higher frequencies. High-quality and compact BPFs are essential for the improvement of the overall performance of an RF receiver [1]. Acoustic-wave resonators (AWR) such as surface acoustic wave (SAW) [2] and film bulk acoustic resonators (FBAR) [3] exhibit remarkable features in the field of integrated circuit design, with excellent quality factor (Q) and compact size. However, the confined electromechanical coupling coefficient of piezoelectric material limits their bandwidth and center frequency expansion, and starting from C-band, the performance of SAW/FBAR decreases sharply in broadband and high frequency [4,5,6], making it unsuitable for high frequency bands.

The substrate integrated waveguide (SIW) is popular, with high-pass characteristics, and it exhibits many advantages, such as low insertion loss, high quality factor, and ease of integration. However, its applications in mmW frequency are limited by the harmonics in stopband and bulk size. Frequency selectivity can be improved by introducing transmission zeros (TZs) into the SIW filter. The filter in [7] employs a source-load coupling to improve the slope of the sideband. The conventional folded coupling configuration in [8] introduces a TZ on each side of the passband. An SIW filter with higher-order mode resonators is proposed in order to achieve modal bypass coupling [9]. Multi-order filters with couplings require complicated coupling structures that are larger in size and create some inevitable losses. Miniaturization is not possible because cascaded multiple resonators prohibit full miniaturization. The use of 3D-stacked structures to achieve miniaturization has also become a popular method in recent years [10,11,12,13,14,15,16]; however, it introduces additional losses.

To avoid the deficiencies mentioned above, a new design structure to implement a quasi-elliptic SIW filter is proposed. Coplanar waveguides (CPW) [17] and complementary split-ring resonators (CSRR) [18,19,20] have been embedded in the SIW cavities to achieve miniaturization, but they result in bad insertion loss. The spoof surface plasmon polariton (SSPP) waveguides that support the surface wave transmission on thin, planar corrugated metals [21,22] have attracted the interest of researchers due to their exceptional properties, such as high confinement [23], low loss [24], potential for solving severe on-chip signal integrity and interference issues [25], as well as the possibility for minimizing the circuit area [26,27]. By embedding the SSPP directly in the SIW, it has been proven to further minimize the filter and improve the transmission performance [28,29]. However, only the upper sideband is improved, due to the characteristics of the SSPPs.

This article proposes a novel SIW-based bandpass filter with periodic arrays of meander-slot etching on the top metallic layer. In [30], an advanced RDL (redistribution layer) process was adopted to design passive components, and it proved to be feasible. In this article, we design the filter using a silicon substrate with 4 RDLs. The dispersion and transmission characteristics are numerically studied by simulation. Due to the distinctive structure of the SSPPs, the asymptotic frequency is lower than that of the original groove structure; only one SIW cavity can achieve the characteristic of multi-order filter, and the size of BPF is greatly diminished. The low-cutoff and high-cutoff frequencies can be flexibly adjusted with the variation of size of the SIW and SSPPs, respectively. With the RDL process, an improved TDC structure is proposed and analyzed. After obtaining the equivalent lump model, a TDC is designed as a part of the coupling circuit to achieve capacitive source-load cross-coupling, and a left transmission zero (TZ) appears successfully. The location of the TZ id can be selected by tuning the TDC and interdigital capacitor. The filter simulation results show that the sideband suppression and stopband suppression are significantly improved, TZ is generated in 37.92 GHz with the rejection of −45 dB, and the 30-dB rectangular coefficient is 1.28.

## 2. Design of the Proposed BPF

**Figure 1:**
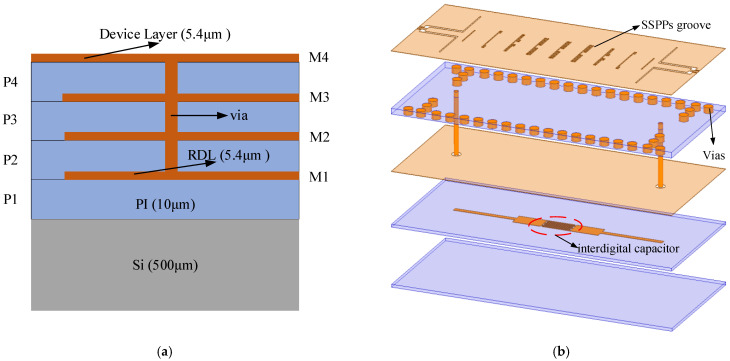
(**a**) Stack-up of the RDL process. (**b**) Configuration of the proposed filter (without Si layer).

This work adopts a redistribution layer (RDL) process whose stack-up is illustrated in Figure 1a. All circuit structures are designed in a four-layer dielectric system interconnected by through-dielectric vias (TDV). The material of the dielectric layer is polyimide (PI-HD4100) with a relative permittivity of 3.2. As shown in Figure 1b, two dielectric layers (P3, P4) are utilized as the SIW cavity. The device layer (M4) is employed to etch the SSPPs grooves and M2 is used as a ground plane. The M1 layer is used to design interdigital capacitors. The low-cutoff frequency is determined by the size of the SIW cavity, and the high-cutoff is finally acquired from the dispersion curve of the SSPPs. All electromagnetic (EM) simulations were carried out using the finite-element method (FEM) of the HFSS 3D simulator.

**Figure 2:**
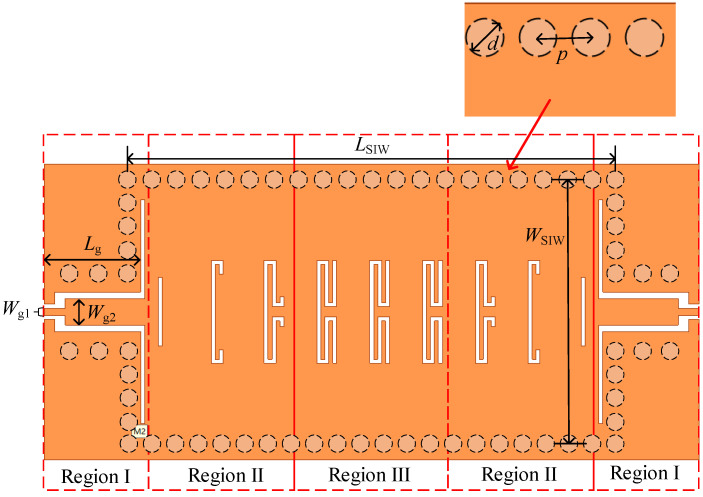
Vertical view of the proposed SIW BPF based on SSPP.

The vertical view of the proposed bandpass filter is shown in Figure 2., An array of periodic curved slots is etched on the top surface metal of the SIW cavity along the direction of electromagnetic wave propagation. The top layer comprises three parts: the GCPW input (region I), the SIW–SSPP transition (region II), and the periodic array part for the propagation of the SSPP. The GCPW transmission line with lower loss and better heat dissipation is more appropriate for mmW integrated circuits; the filter is excited by two 50-Ω GCPW transmission lines with a pair of quarter wavelength coupling slots. Three curved slot cells of different lengths ($L_{s1}$, $L_{s2}$, $L_{s3}$), which are gradually increased in length, constitute region II. The increase is optimized to achieve smooth transition and mode matching between the SIW and SSPP, and then the SSPP mode is effectively excited. The dimensions of the BPF are calculated below.

The TE101 mode resonant frequency of the SIW cavity is adopted as the low-cutoff frequency, and the dimensions are determined as in [31]:

(1) $$f_{0}=\frac{c_{0}}{2\sqrt{\varepsilon_{r}}}\sqrt{\frac{1}{W_{SIW}^{2}}+\frac{1}{L_{SIW}^{2}}}$$

where $W_{SIW}$ and $L_{SIW}$ are the width and length of the equivalent rectangular waveguide, calculated as:

(2) $$W_{SIW}=w-1.08\frac{d^{2}}{p}+0.1\frac{d^{2}}{w}$$

(3) $$L_{SIW}=l-1.08\frac{d^{2}}{p}+0.1\frac{d^{2}}{l}$$

where w and l represent the width and length of the SIW cavity, respectively, d is the diameter of Cu via, and p is the center-to-center pitch between the adjacent via holes. $c_{0}$ is the light velocity in vacuum, and $\varepsilon_{r}$ is the relative permittivity. In order to make the resonance frequency $f_{0}=$ 47 GHz, $W_{SIW}=$ 2 mm and $L_{SIW}=$ 3.8 mm is finally determined by the above equations.

**Figure 3:**
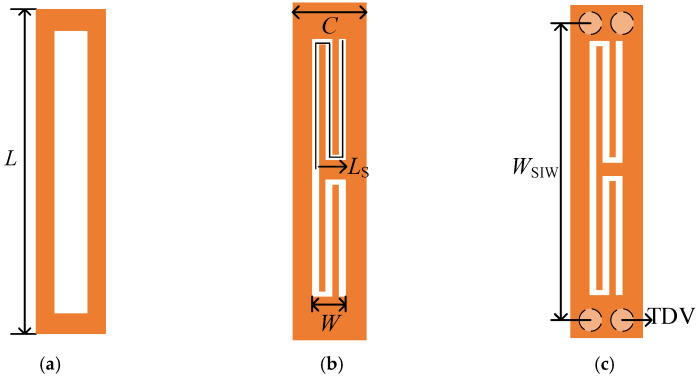
The vertical view of (**a**) rectangular groove unit cell, (**b**) meander–slot unit cell, (**c**) SIW with meander-slot unit cell.

The schematic diagram of the unit cell of the SSPP is illustrated in Figure 3. According to [32], the dispersion curve for the SSPP mode propagated in the metallic grove array can be expressed as

(4) $$k=k_{0}\sqrt{1+\frac{W^{2}}{C^{2}}tan^{2}\left(k_{0}L\right)}$$

where $k_{0}=2\pi /\lambda$ refers to the propagation constant in free space, C is the width of the unit cell, W and L represent the width and depth of the grooves, respectively.

When the width of the groove W and the width of the unit cell C is fixed, the depth of the groove L is the main factor affecting the dispersion. Meanwhile, to keep the occupied area of the groove unchanged, a meander-slot structure is proposed. Figure 3a,b show a traditional groove unit and a meander-slot unit with the same height (L). Figure 3c shows the unit cell consisting of an SIW and meander-slot units. The orange and white parts represent the metal surface on the top layer and the slot line, respectively. The geometric parameters are set as shown in Table 1.

**Figure 4:**
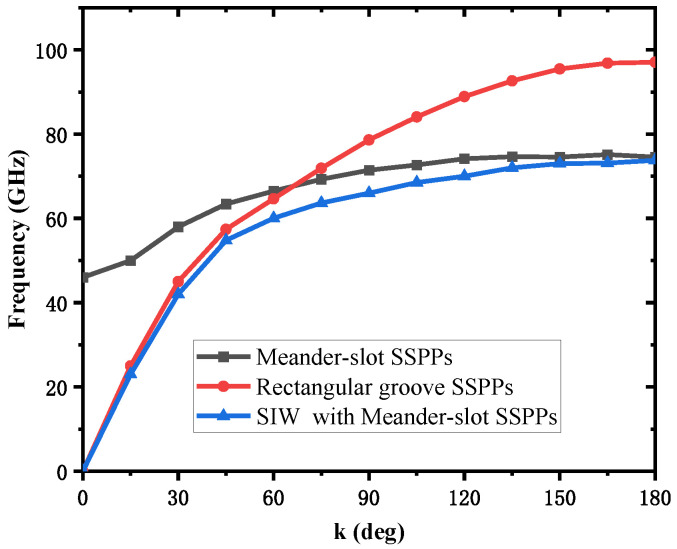
Dispersion curve of the unit cell.

To visualize the relationship between the dimension and frequency of the SSPP, the dispersion curves are calculated using the eigenmode solver of commercial electromagnetic software. In the simulations, the dispersion relation was obtained by calculating the eigenfrequency of the SSPPs unit. Figure 4 shows the dispersion curves of fundamental SSPP modes, k represents propagation constant, and k is swept from 0° to 180° between the period boundaries in the propagation direction. It is clear that the two dispersion curves have similar frequency trends, increasing as k increases. It is obvious that these two dispersion curves have similar frequency variation trends, but the asymptotic frequency of the groove unit is 97 GHz when the asymptotic frequency of meander-slot unit is 73.7 GHz. This means that adopting a meander-slot SSPP in the same occupied area can reduce the available asymptotic frequency, which is effective in minimizing the device. Figure 4 demonstrates that the SIW can be combined with SSPPs to achieve bandpass characteristics, starting from the cutoff frequency, and its dispersion curve behaves like the SIW in the low frequency range and like SSPPs in the high frequency range.

**Figure 5:**
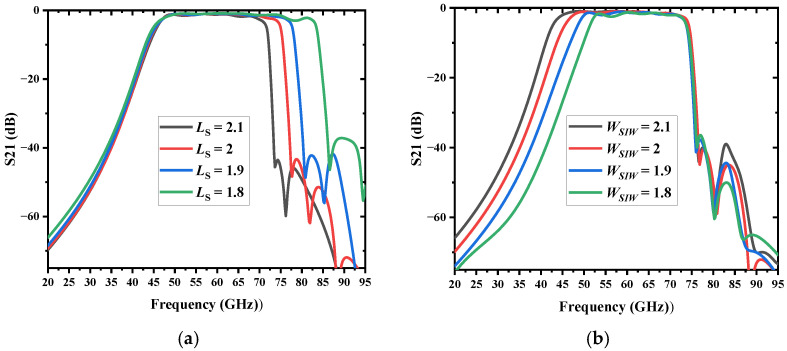
Simulated results of the proposed bandpass filter with different (**a**) SSPPs length $L_{S}$. (**b**) SIW width $W_{SIW}$.

To confirm the passband alteration characteristics of the proposed filter, a parameter inquiry was conducted. The simulated results of transmission coefficients are demonstrated in Figure 5 with different geometric parameters. It can be inferred that the high-cutoff frequency of the proposed filter is determined by the SSPPs length $L_{s}$, and the low-cutoff frequency is determined by the SIW width $W_{SIW}$. Figure 5a shows that when the value of $L_{S}$ increases gradually, the upper sideband frequency of the filter will move to the left, while the lower sideband remains constant; the bandwidth becomes narrower accordingly. It can be attributed to the gradual increase of the propagation constant and momentum, and the decrease of the asymptotic frequency, with the increase of the geometric length $L_{s}$ of the curved slot. In addition, as shown in Figure 5b, the $W_{SIW}$ decreases and the lower sideband shifts to the right because the resonant frequency of the SIW is determined by the SIW width $W_{SIW}$. Therefore, the bandwidth of the filter can be flexibly controlled by adjusting the size of the SIW cavity and the SSPP slot. Finally, the dimensions of the BPF are determined by the low-cutoff frequency (47 GHz) and high-cutoff frequency (74 GHz), as shown in Table 2.

**Figure 6:**
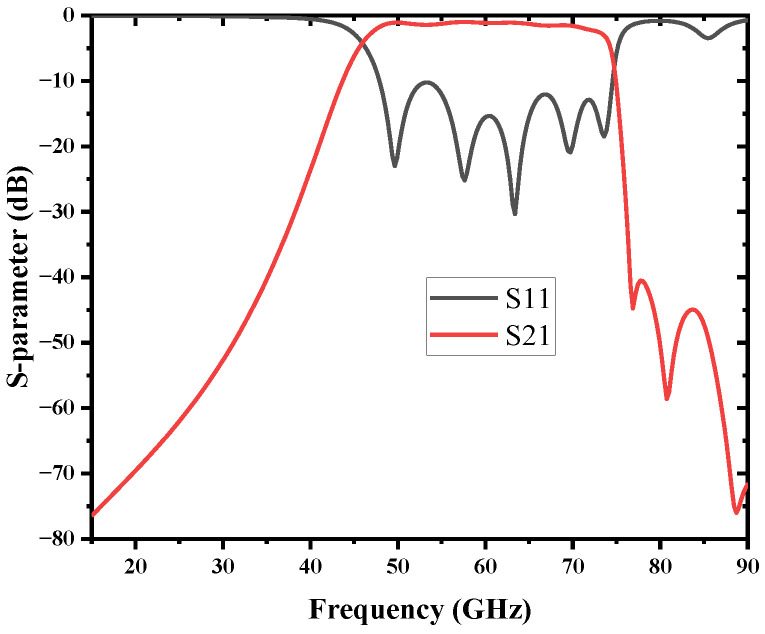
Simulation results of the BPF based on SIW and SSPP.

The SIW filter using SSPP without source-load coupling was simulated, and the result is shown in Figure 6. Good bandpass features and high-efficiency propagation are obtained. The BPF operates from 46.13 GHz to 74 GHz; the 30-dB bandwidth is from 37.5 GHz to 76.5 GHz; and the corresponding rectangular coefficient is 1.4.

**Figure 7:**
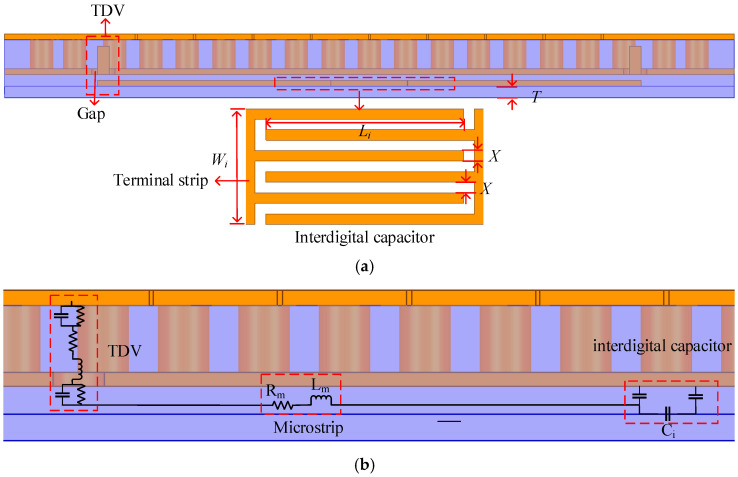
(**a**) Cross-section schematic view of the filter; (**b**) Equivalent lump model of this proposed coupling circuit (**left**).

With the multi-layer stacked structure, 3D interconnections can be applied. To further improve the performance of the filter, source-load cross-coupling was employed. The sectional drawing of the proposed filter with source-load cross-coupling is shown in Figure 7a. With the adoption of the TDC, signals can be transmitted vertically through different layers. There are two microstrip lines and an interdigital capacitor in the M1 layer. The interdigital capacitor is more suitable for applications where low values of capacitance are required. Letting the finger width (*X* = 0.025 mm) equal the slot width to achieve maximum capacitance density, the expression [33] for estimation of capacitance of the interdigital capacitor can be given by

(5) $$C_{i}=\left(\varepsilon_{r}+1\right)\frac{L_{i}}{W_{i}}\left[\left(n-3\right)A_{1}+A_{2}\right]$$

where *n* is the number of fingers and *C* is in pF. $A_{1}=$ 0.75 and $A_{2}=$ 0.175 are determined by the value of $\frac{T}{X}$ refer to in [33], where *T* is the height of the substrate (T= 0.01 mm). As shown in Figure 7b, the complete coupling circuit consists of two TDCs, two microstrips and an interdigital capacitor which are lumped models. The collaborative design method of the TDC and the coupling circuit can be used to minimize the influence of the interconnection structure. The series connection of the interdigital capacitor and TDC can also reduce the circuit capacitance and increase the adjustment range of the interdigital capacitor. The admittance of the equivalent coupling circuit with the TDC in Figure 7b can be expressed as

(6) $$Z=Z_{t}+R_{m}+j(\omega L_{m}-\frac{1}{\omega C_{i}})$$

where $Z_{t}$ is the impedance of the TDC structure, which is analyzed below.

**Figure 8:**
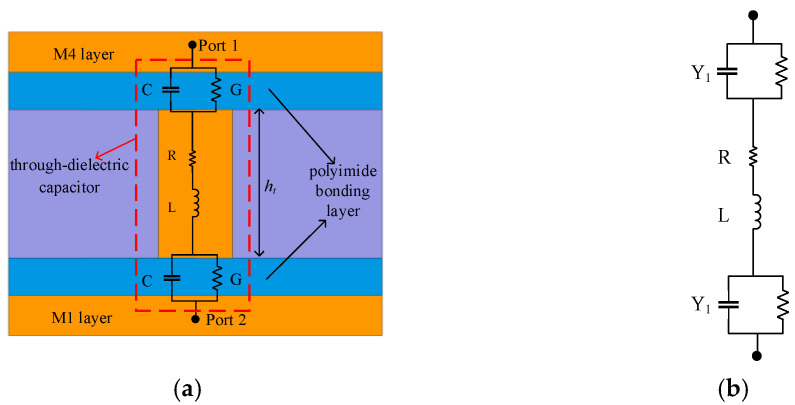
Structure of TDC in the designed filter: (**a**) Cross-section schematic view with the main parasitic components; (**b**) Equivalent lump model.

Figure 8a shows the structure and the main parasitic components of the TDC in the designed filter. Between the two metal layers, two polyimide bonding layers act as capacitive coupling media, while one is filled with copper through the dielectric via. The equivalent lump model of the TDC can be referred to [34], as shown in Figure 8b.

The impedance $Z_{t}$ can be approximately expressed by

(7) $$Z_{t}=\frac{2}{Y_{1}}+j\omega L+R$$

(8) $$Y_{1}=G+j\omega C$$

where $Y_{1}$ is the admittance of the coupling medium, *R* and *L* represent via resistance and inductance, respectively, when *C* and *G* represent the parasitic capacitance and conductance, respectively.

**Figure 9:**
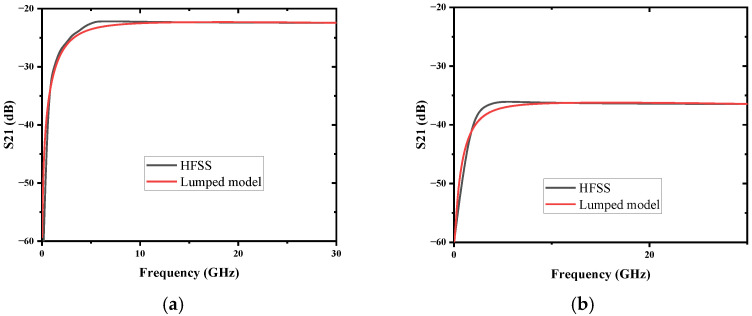
Comparison of S parameters between TDC and model with different $h_{t}$: (**a**) $h_{t}=$ 0.024 mm; (**b**) $h_{t}=$ 0.08 mm.

To accurately verify the compatibility and analyze the electrical performance of the TDC, Ansys Q3D was used to extract all the parasitic components of TDC. Substituting all the parasitic components into the proposed lump model then enables the transmission characteristics to be obtained by the ADS. The comparison of transmission characteristics between the TDC simulation result by HFSS and the proposed lumped model is shown in Figure 9. Two experimental cases with different heights of through dielectric via ($h_{t}$) are used in simulation. The lumped models of the two cases both match well with the HFSS simulations. The insertion loss S_21_ of TDC increases with frequency below 10 GHz, which implies that the coupling behavior of the TDC is mainly capacitive coupling, and slightly decreases in high frequency. The capacitive TDC effect is dominant in the lower frequency range, and the inductive TDV effect becomes dominant in the higher frequency range [34]; the inductance of the TSV channel starts to affect the insertion loss over 10 GHz. The above analysis shows that the proposed TDC can be regarded as a CGRL lumped model, collaboratively designed with the coupling circuit. In addition, it will not seriously influence the circuit during the working frequency.

**Figure 10:**
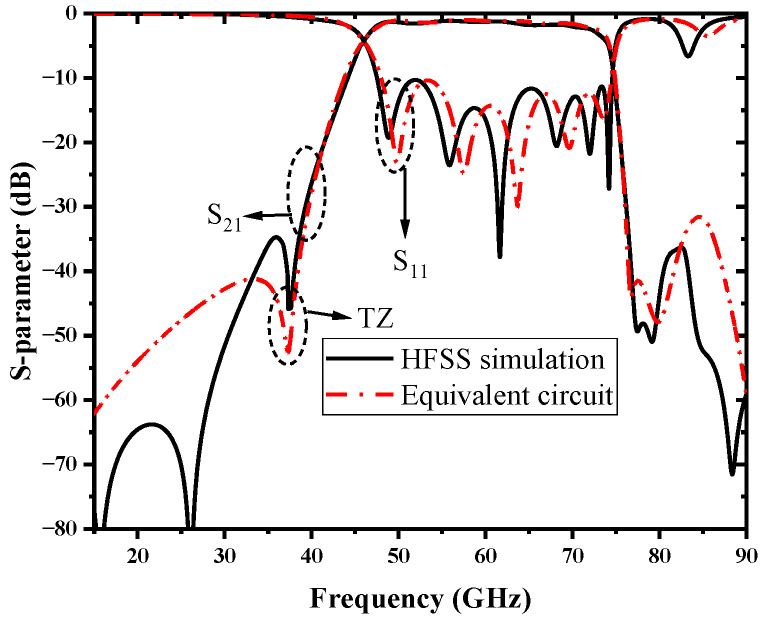
Comparison of equivalent circuit and filter simulations.

To further verify the lumped model of the whole coupling circuit, the S-parameter is extracted from Figure 6 and simulated with the above coupling circuit model, and the result is compared with the HFSS simulation of the proposed filter, as shown in Figure 10. The frequency responses of the equivalent circuit and filter simulations both exhibit a transmission zero at 37.6 GHz, and their simulation results are in good agreement.

According to the above analysis and verification, the proposed TDC structure can be applied to transmit signals vertically through different layers, and it is possible to design a circuit with a lumped model. With the adoption of the previous lumped model, capacitive source-load cross-coupling is investigated.

**Figure 11:**
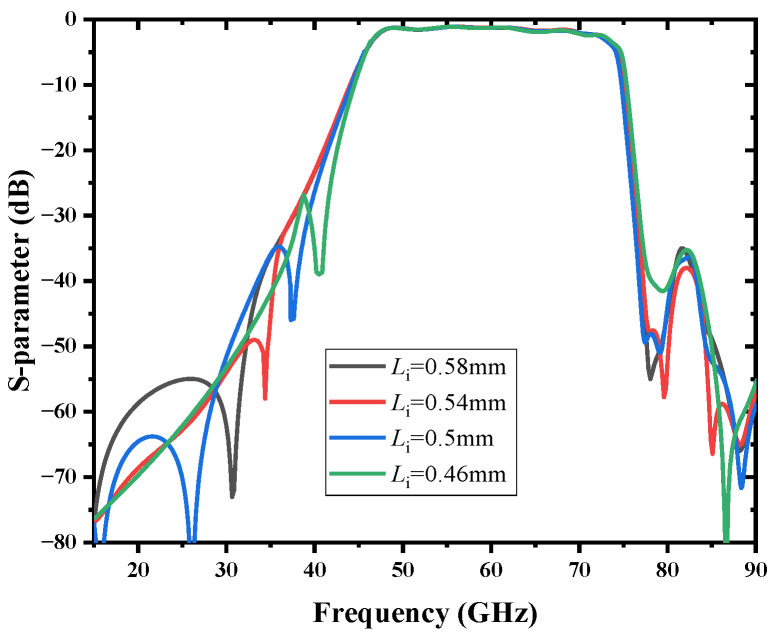
Simulated results with different interdigital capacitor length $L_{i}$.

The capacitance of the interdigital capacitor will change over $L_{i}$. By adjusting the capacitance of the interdigital capacitor, the location of the TZ is moved, as shown in Figure 11, and the suppression deteriorates as TZ approaches the passband. To optimize the performance of the filter, a transmission zero is finally generated at 37.92 GHz, and the height of the through dielectric via $h_{t}=$ 0.0246 mm and the radius of the via $r_{t}=$ 0.04 mm are finally determined.

## 3. Results and Analysis

**Figure 12:**
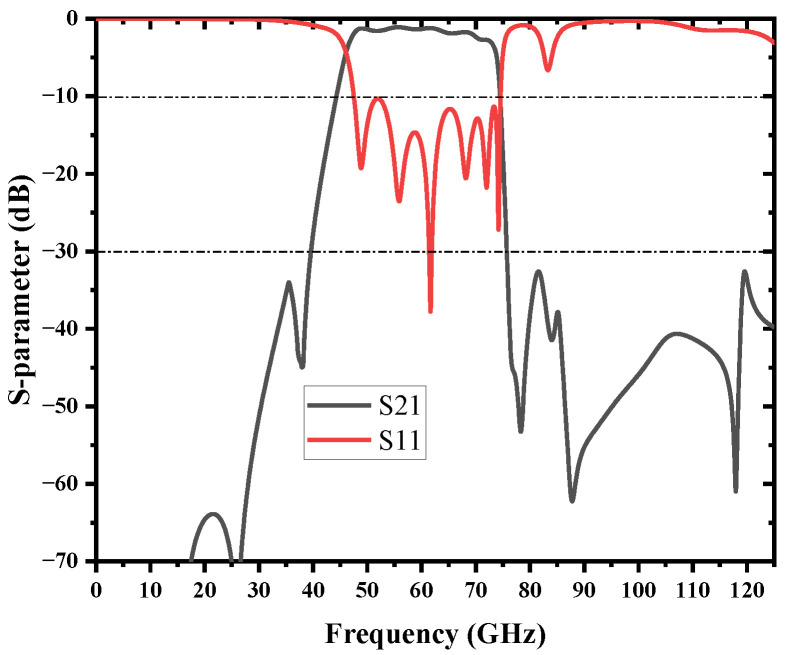
Simulation result of the bandpass filter with source-load coupling.

The simulated results of the S-parameters with reflection coefficients (S11) and transmission coefficients (S21) are demonstrated in Figure 12. An SIW cavity is employed to provide multi-transmission poles in order to achieve a passband. The compact BPF operates at 46.1–73.7 GHz with a wide 3 dB FBW of 45.8%. Good bandpass features and high-efficiency propagation are obtained; the minimum insertion loss is 1.08 dB, and the return loss is better than −10 dB in the passband. Due to the steep upper sideband, the 30-dB bandwidth is from 40 GHz to 75.4 GHz, and the corresponding rectangular coefficient is 1.28 when the BPF without TZ is 1.4. Meanwhile, the stopband rejection is better than 30 dB up to 125 GHz (2$f_{c}$). The final core size of the BPF is 2 mm × 4.4 mm (0.74$\lambda_{g}$ × 1.63$\lambda_{g}$). It is notable that the proposed BPF has the merits of competitive wideband performance, broad stopband, and compact size. The performance of the proposed filter is compared with other work in Table 3; all the data are based on the simulation results.

## 4. Conclusions

In this article, an SIW-based 46.1 GHz to 73.7 GHz bandpass filter is proposed, which is designed on the RDL process. The simulation results show low insertion loss, good frequency selectivity, and wideband harmonic suppression. The etching of periodic arrays of meander slot units on the top metallic layer enables the substrate integrated waveguide (SIW) to attain bandpass characteristics with high-efficiency and strongly confined microwave SSPP transmission. In addition, its asymptotic frequency can be significantly reduced compared to the conventional groove SSPP. This means that the propagation of the structure can occupy a smaller area with the benefit of lower cost, especially for the process of integrating a passive device, and the leakage loss will decrease as the gap area reduces. The simulated results show that the bandwidth of the proposed filter can be flexibly elected by tuning the geometric parameters of the SIW and SSPPs. To improve the performance of the upper sideband, a novel 3D capacitive interconnection is proposed and investigated, the lump model of the TDC is obtained, and a collaborative design with interdigital capacitance is adopted to achieve a transmission zero. By adjusting the height of the metal via ($h_{t}$) and the capacitance of the interdigital capacitor ($C_{i}$), the location of the TZ can be selected. The proposed filter based on one SIW resonator is the same as the multi-order filter with coupling, realizing the miniaturization with good performance.

## Figures and Tables

**Table 1:** The geometric parameters of the SSPPs unit cell.

*C*	*L*	*L_s_*	*W*	*W_SIW_*
0.4 mm	0.39 mm	1 mm	0.075 mm	2 mm

**Table 2:** The dimensions of the BPF.

*L_SIW_*	*W_SIW_*	*L_g_*	*W_g1_*	*W_g2_*	*L_s1_*	*L_s2_*	*L_s3_*
4 mm	2 mm	0.76 mm	0.055 mm	0.2 mm	0.26 mm	0.49 mm	0.78 mm

**Table 3:** Performance comparisons of BPFs operating above millimeter-wave. (All the data are based on the simulation results).

Ref.	$f_{c}\;(\mathrm{GHz})$	$\mathrm{Size}\;(\lambda_{g}$ $\;\times \;\lambda_{g})$	MIN. IL (dB)	$\frac{BW_{30\mathrm{dB}}}{BW_{3\mathrm{dB}}}$	**FBW (%)**
[4]	27	0.09 × 0.09	0.84	2.6	20
[6]	3.5	2.04 × 0.85	1.139	1.25	56
[7]-2	9.1	0.218 × 0.218	0.84	2.5	19.8
[9]	93	2.31 × 1.57	4	1.59	3.5
[29]	236.5	1.16 × 0.37	2	1.86	12
This work	60	1.63 × 0.74	1.08	1.28	45.8
